# Homology directed correction, a new pathway model for point mutation repair catalyzed by CRISPR-Cas

**DOI:** 10.1038/s41598-022-11808-2

**Published:** 2022-05-17

**Authors:** Brett M. Sansbury, Amanda M. Hewes, Olivia M. Tharp, Sophia B. Masciarelli, Salma Kaouser, Eric B. Kmiec

**Affiliations:** 1 Gene Editing Institute, ChristianaCare Health System, 550 S College Ave, Suite 100A, 2nd Floor, Newark, DE 19713 USA; 2 Department of Medical and Molecular Sciences, University of Delaware, Newark, DE USA

**Keywords:** Genetics, DNA mismatch repair, Molecular biology

## Abstract

Gene correction is often referred to as the gold standard for precise gene editing and while CRISPR-Cas systems continue to expand the toolbox for clinically relevant genetic repair, mechanistic hurdles still hinder widespread implementation. One of the most prominent challenges to precise CRISPR-directed point mutation repair centers on the prevalence of on-site mutagenesis, wherein insertions and deletions appear at the targeted site following correction. Here, we introduce a pathway model for Homology Directed Correction, specifically point mutation repair, which enables a foundational analysis of genetic tools and factors influencing precise gene editing. To do this, we modified an in vitro gene editing system which utilizes a cell-free extract, CRISPR-Cas RNP and donor DNA template to catalyze point mutation repair. We successfully direct correction of four unique point mutations which include two unique nucleotide mutations at two separate targeted sites and visualize the repair profiles resulting from these reactions. This extension of the cell-free gene editing system to model point mutation repair may provide insight for understanding the factors influencing precise point mutation correction.

## Introduction

A major goal of gene therapy is to edit and correct a genetic mutation within the context of the human chromosome. While foundational work began years ago^1^, the breakthrough CRISPR-Cas technology has increased optimism that genetic repair of point mutations might finally become a clinical reality. The straightforward therapeutic vision of direct gene repair is to resolve genetic disorders caused by point mutations, such as Sickle Cell Disease (SCD). While the “surgical” form of gene repair is clearly a direct path to treatment and cure, the current landscape of scientific work within the field, with respect to precise gene correction, remains unclear and full of significant barriers. The most prominent challenge centers on the diversity of genetic outcomes that have resulted from sophisticated attempts to correct a single base mutation using CRISPR-based gene editing. Our work^2–4^ and others^5–7^ have revealed that while successful point mutation repair can be achieved, it is almost always associated with a significant amount of imprecise editing, particularly at the targeted sites where the CRISPR-Cas complex was designed to act, i.e., on-site mutagenesis*.*

Our laboratory has previously worked to investigate precise point mutation correction of the single base mutation responsible for SCD using a CRISPR-Cas9 ribonucleoprotein (RNP) complex and a single-stranded oligonucleotide (ssODN) donor repair template^2^. We were among many laboratories that analyzed the effectiveness of gene editing at the beta globin locus in somatic cells and clearly demonstrated single base conversion at the targeted site. While successful single base mutation correction was observed, unintended on-site changes were also found at an alarmingly high rate. We have also confirmed other laboratories initial observations that unintended genetic changes were prevalent after CRISPR-Cas gene editing in CD34 + cells^4,8,9^.

To address these challenges, many laboratories focus on improving the precision and activity of the CRISPR-Cas system through genetically reengineering the Cas protein^10–13^ or exploring the possibility of altering the chemical composition and molecular features of the DNA repair templates^14–16^. Significant improvement in efficiency and precision has not yet been sufficiently realized which could explain, in part, why many clinical trials are not aiming to repair point mutations within the context of the chromosome. For SCD, clever workaround strategies for the reactivation of fetal hemoglobin (HbF) expression to counteract the effects of this disease, are becoming prevalent^17–19^. An innovative approach being undertaken for Hereditary Transthyretin Amyloidosis (hATTR) avoids correction, but rather aims to disable gene expression, so that patients fail to produce the faulty protein altogether^20^. A similar strategy is being used for CRISPR-directed gene editing of solid tumors^21^. Research groups have also used ex vivo approaches for certain cancers, harvesting patient T-cells and targeting with CRISPR-Cas before re-introducing the edited population back into the patient^22^.

Our laboratory has employed a different strategy to understand point mutation repair, focusing on developing models to study and understand the biochemical and molecular factors that influence precise and imprecise gene editing. Previously, we established several foundational gene editing reaction model systems to examine and elucidate the molecular mechanisms and regulatory circuitries that surround CRISPR-directed gene editing in mammalian cells. We reported the development of a cell-free system and successfully recapitulated a group of CRISPR-directed gene editing reactions including DNA insertion, gene segment replacement and site-specific deletion^23–25^. To a large extent, this system maintains the microenvironment in which both non-homologous end joining (NHEJ) and homology directed repair (HDR) pathways are free to operate, enabling methodical manipulation of individual reaction components. These model reactions have allowed us to identify dominant pathway(s) that regulate CRISPR activity, compare gene editing activity between various Cas proteins, measure gRNA heterology tolerance for potential off-target cleavage, identifying the optimal structure and characteristics of donor templates that can help direct DNA repair and elucidating the factors that influence the balance between NHEJ and HDR^23–27^. These discoveries have helped provide clarity surrounding reaction mechanics and guide strategies designed to improve gene editing precision.

It is now widely known that with the present array of gene editing tools, the genetic outcome of point mutation repair, in somatic or progenitor cells, is still far from precise. Imprecise editing leaves a genetic footprint that could ultimately lead to changes in gene expression and negatively impact and influence cell metabolism and/or growth. It has been stated that point mutation correction of the SCD mutation is termed clinically significant when correction efficiency is at least 20%^28^, so while point mutation repair efficiency is not needed to be incredibly high, it does need to be precise. Thus, a translational model system to evaluate regulatory factors and approaches to increase the precision of such reactions should be developed and utilized. Here, we take a reductionist approach and expand the utility of the cell-free extract system by generating a model for biochemical enabling, methodical molecular analyses of Homology Directed Correction (HDC), point mutation repair, and creating a new tool for identifying important regulatory factors that control the efficiency and precision with which point mutations are repaired.

## Results

**Figure 1:**
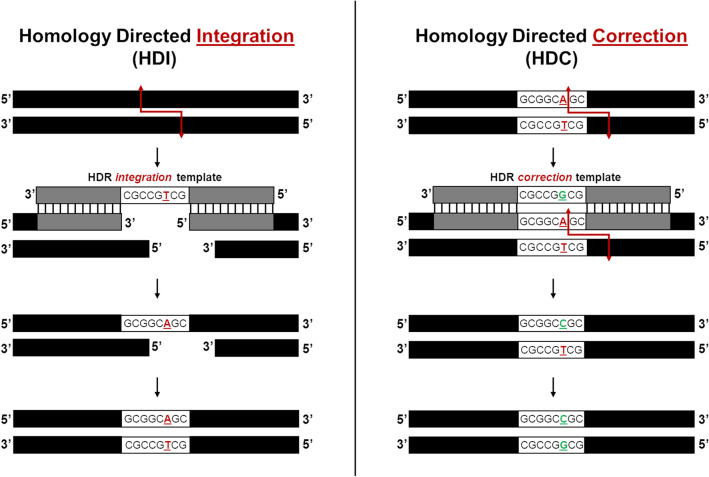
Homology Directed Integration (HDI) and Homology Directed Correction (HDC) reactions. Illustrations show the HDR *integration* template-directed HDI reaction used to generated NotI-MT plasmids (left) and HDR *correction* template-directed HDC reaction used for point mutation repair of NotI-MT plasmids (right).

**Figure 2:**
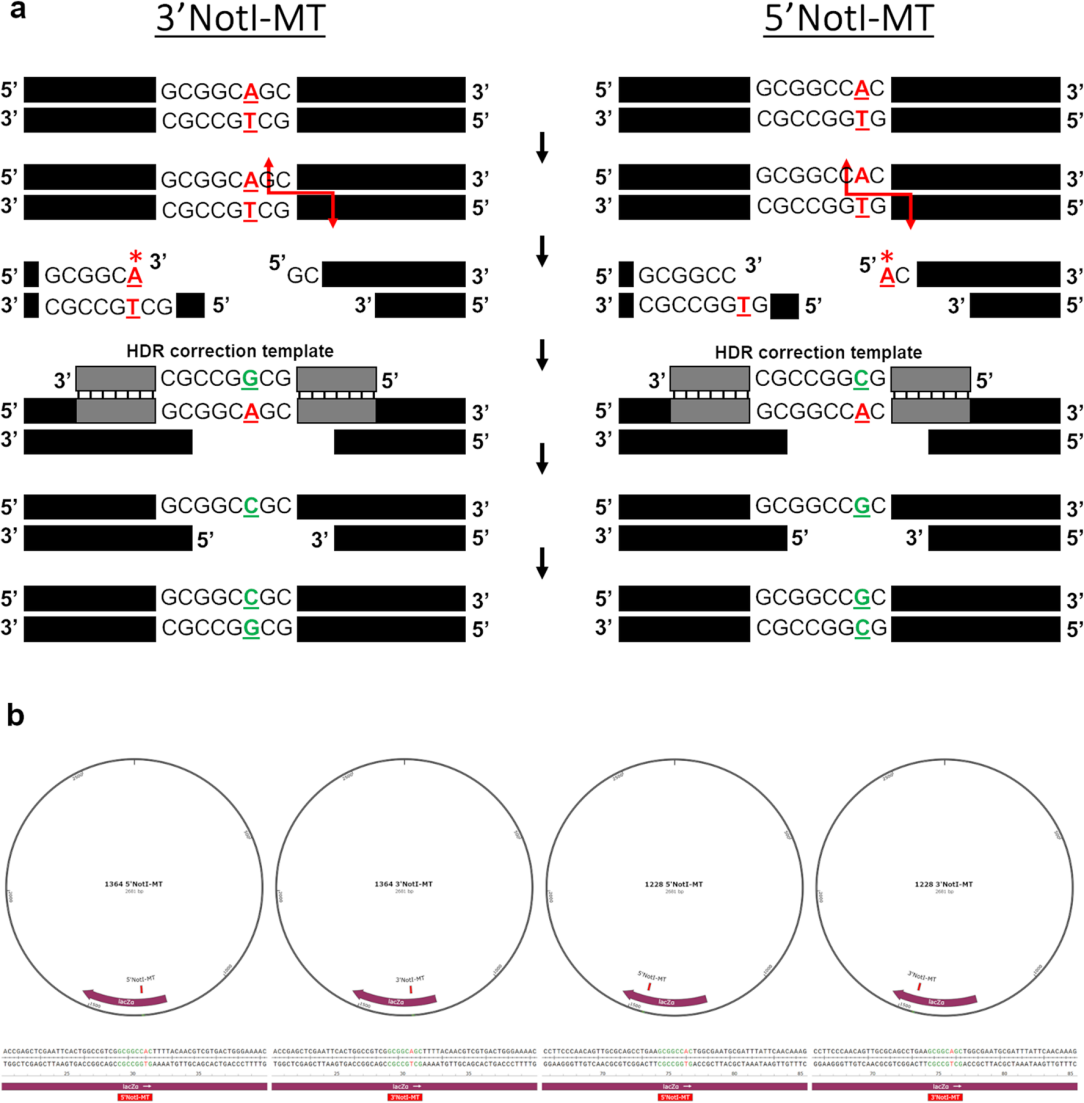
HDC reactions and NotI-MT plasmids. (**a**) Illustrations are shown of the HDC reactions used for the single base point mutation repair of 3′NotI-MT A > C (left) and 5′NotI-MT A > G (right). (**b**) Representations are shown of the four NotI-MT plasmids generated and the unique mutations incorporated, 5′ or 3′, at each of the two unique targeted sites, 1364 or 1228.

To distinguish point mutation repair, we define fragment insertion as Homology Directed Integration (HDI) and single nucleotide exchange, or point mutation repair, as Homology Directed Correction (HDC). We established the HDC model by utilizing the standard HDI reaction described in our previous work^23–26^, with CRISPR-Cas12a complexes and ssODN HDR *integration* templates, as shown in Fig. 1 (left). We began by targeting a plasmid, pHSG299, which does not contain a native NotI restriction enzyme site and generated four unique NotI mutated plasmids (NotI-MTs). These mutated plasmids included two unique single nucleotide mutations in the newly added NotI sites, at two locations along the targeted *lacZ* gene. These four mutated plasmids would then serve as our targets for HDC reactions, as shown in Fig. 1 (right). Cas12a was the optimal choice for this CRISPR-Cas targeting, as Cas9 would not have been able to re-target the mutated site of interest, as the integration would happen 3 bps proximal to the Cas9 PAM site. Cas12a, on the other hand, cuts distal to the PAM site, 18 and 23 bps, and can easily re-target the site after the integration. The mutated NotI sites embedded in the 3′NotI-MT and 5′NotI-MT plasmids are 5′-GCGGC**A**GC-3′ and 5′-GCGGCC**A**C-3′, respectively, termed relative to the exposed termini generated by Cas12a cleavage (Fig. 2a). The four new plasmids, 1364 3′NotI-MT, 5′NotI-MT and 1228 3′NotI-MT, 5′NotI-MT, differed only in the relative locations along the *lacZ* gene, 1364 or 1228, and the position of the mutated nucleotide within the NotI site, 3′ or 5′ (Fig. 2b). The ssODN HDR *integration* templates that were used to generate these NotI mutant plasmids, contained either the 3′ or 5′ mutations, are shown in Supplemental Fig. 1A, B. Each *integration* template was 70 bases in length and contained a mutated eight-base NotI restriction enzyme site in the center and flanked by homology arms upstream and downstream from the cut sites. After HDI reactions were completed, DNA sequencing of the newly generated NotI mutant plasmids confirmed proper integration and positioning of the mutated bases in the constructs (Supplemental Fig. 1C–D).

**Figure 3:**
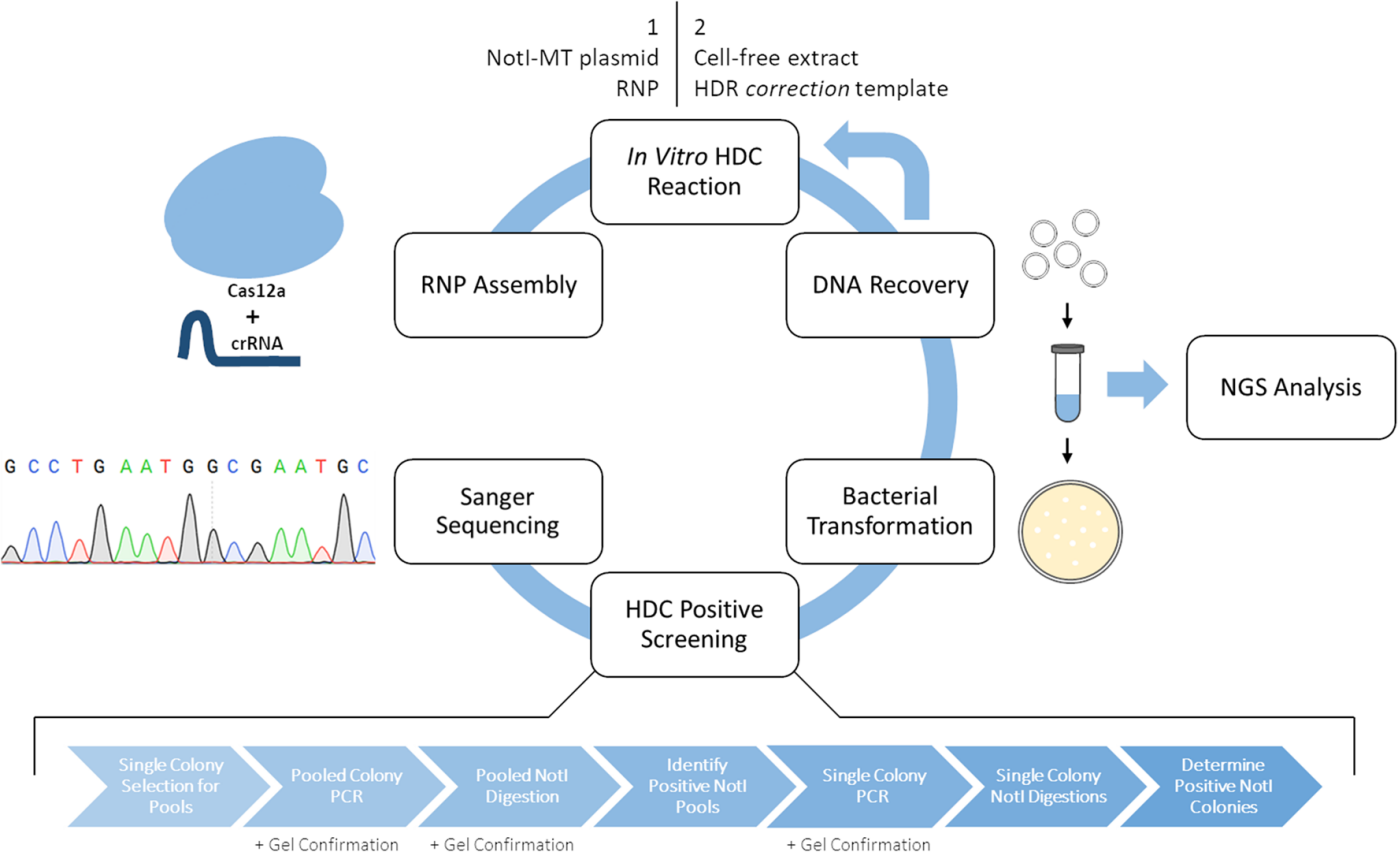
HDC reaction experimental workflow, screening and analysis. A Cas12a RNP complex is assembled and added to the in vitro cleavage reaction with a NotI-MT plasmid. DNA is then recovered and added to the in vitro re-circularization reaction with the CFE and HDR *correction* template, which will facilitate point mutation repair. DNA is then recovered and transformed into competent *E. coli.* Successful HDC reactions were then identified through a series of NotI digestion screenings, as well as DNA sequencing analysis via Sanger sequencing of transformed bacterial clones and NGS of DNA recovered after HDC reactions.

An overview of the experimental workflow carried out for the HDC reactions following the construction of the NotI mutated plasmids is outlined in Fig. 3. Each NotI-MT plasmid was individually targeted by a Cas12a RNP complex to initiate the single base mutation repair, HDC reaction. Once cleavage had been initiated, a 70-base ssODN HDR *correction* template was introduced to direct repair; this template contains the normal NotI restriction site (3′-CGCCG**GC**G-5′) with sequence complementary both upstream and downstream from the 1364 or 1228 targeted sites. These donor templates, coupled with the addition of an HEK-293 cell-free extract (CFE), directed correction of the 3′NotI-MT (A–C) and 5′NotI-MT (A–G) mutations. NotI enzyme digestion and gel electrophoresis were used as an initial screening assay to assess the efficiency of point mutation correction from pooled populations of bacterial colonies transformed with plasmid recovered from HDC reactions. Once NotI digestion positive pools were identified, clones from the positive pools were subjected to individual colony PCR and NotI digestions. While the efficiency of correction varied between the 3′NotI-MT and 5′NotI-MT reactions at the 1364 site, successful repair was detectable at both 1228 and 1364.

**Figure 4:**
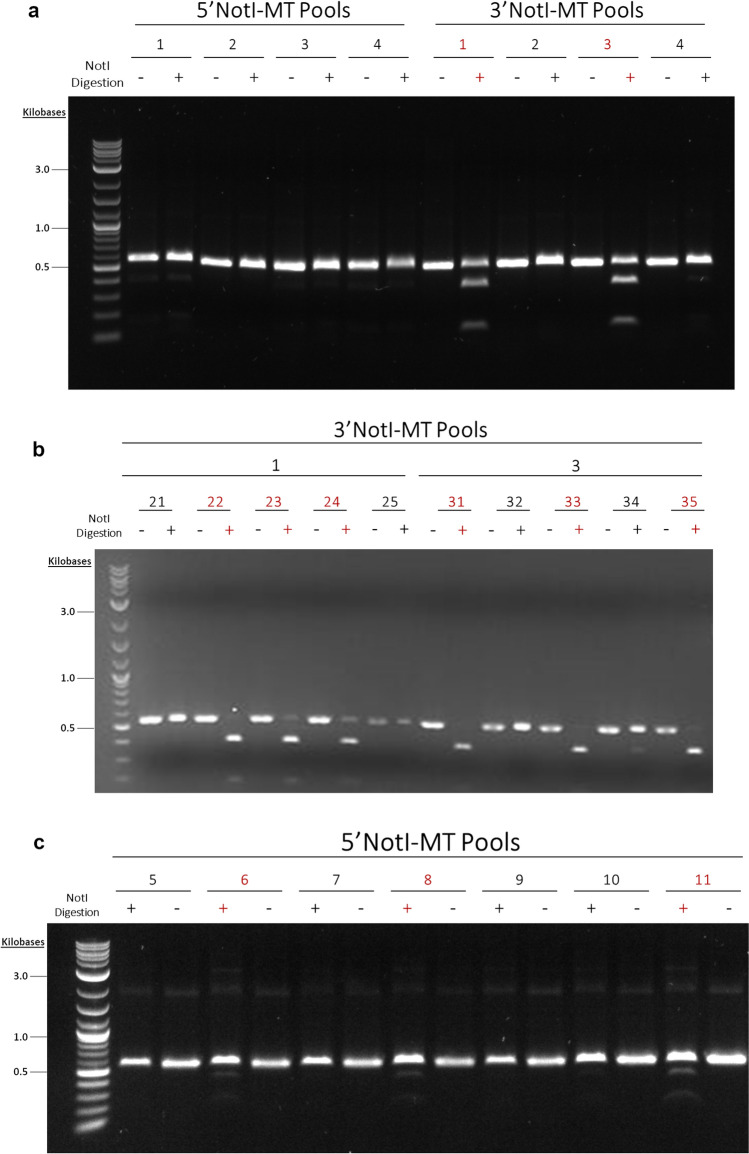
Pooled and single colony NotI digestions of 1364 NotI-MTs. (**a**) NotI digestions were done on four 5′ and 3′NotI-MT colony PCR pools consisting of 5 single colonies per pool. (**b**) NotI digestions were done on individual colonies from 3′NotI-MT colony PCR Pools 1 and 3. (**c**) NotI digestions were done on seven additional 5′NotI-MT colony PCR pools consisting of 5 single colonies per pool. Pools containing uncorrected NotI-MT DNA can be seen in lanes containing intact, undigested linear PCR amplicons as single bands. Pools containing corrected NotI-MT DNA can be seen in lanes with multiple bands after amplicon digestion are shown in red, with the upmost showing uncut, uncorrected PCR amplicon and the lower two showing successful NotI digested fragments. Original gels are presented in Supplementary Fig. 2.

Our initial screen of the 1364 3′NotI-MT and 5′NotI-MT correction reactions included four colony PCR pools for each NotI-MT variant, where each pool consisted of five randomly selected single colonies (Fig. 4a). This screening revealed that two of the four 3′NotI-MT pools, Pool 1 and Pool 3, revealed PCR amplicon digestion, confirming the presence of corrected NotI sites within the pooled populations (Fig. 4a, right). To further screen NotI digestion positive pools, NotI digestions were carried out on all ten individual colonies comprising the populations of both Pool 1 and Pool 3 (Fig. 4b). Three of the five colonies present in both 3′NotI-MT Pools 1 and 3 contained DNA templates which had been corrected. While 3′NotI-MT correction was readily observable at the 1364 site in the initial screening process, we did not see evidence of positive NotI digestion in the initial four 5′NotI-MT pools (Fig. 4a, left). To investigate this apparent difference in correction efficiency between the 3′NotI-MT and 5′NotI-MT pools at the 1364 site, an expanded NotI digestion screening was carried out on seven additional 5′NotI-MT colony pools, each pool consisting of five randomly selected colonies (Fig. 4c). From this second round of 5′NotI-MT screening, three of the pools exhibited low but observable NotI digestion, suggesting that some degree of correction was present within the pooled populations.

**Figure 5:**
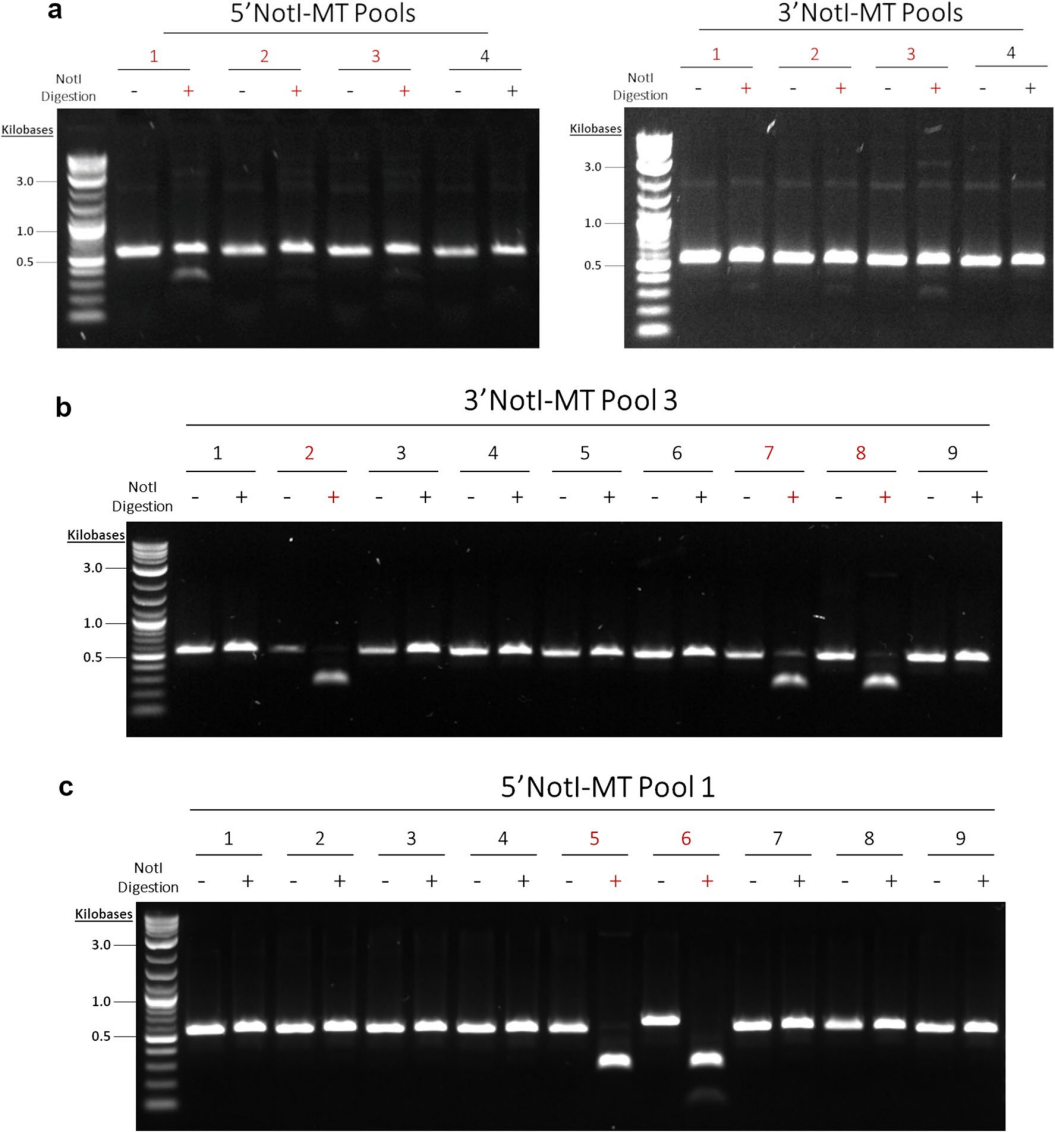
Pooled and single colony NotI digestions of 1228 NotI-MTs. (**a**) NotI digestions were done on four 5′ and 3′NotI-MT colony PCR pools consisting of 9 single colonies per pool. (**b**) NotI digestions were done on individual colonies from 3′NotI-MT colony PCR Pool 3 and (**c**) 5′NotI-MT colony PCR Pool 1. Pools containing uncorrected NotI-MT DNA can be seen in lanes containing intact, undigested linear PCR amplicons as single bands. Pools containing corrected NotI-MT DNA can be seen in lanes with multiple bands after amplicon digestion are shown in red, with the upmost showing uncut, uncorrected PCR amplicon and the lower two showing successful NotI digested fragments. Original gels are presented in Supplementary Fig. 3.

We then utilized the same screening workflow described above for assessing the 1228 3′NotI-MT and 5′NotI-MT correction reactions. Our initial screen here also included four colony PCR pools for each mutation variant, where each pool consisted of nine randomly selected single colonies (Fig. 5a). Unlike the differences seen in the 1364 3′NotI-MT and 5′NotI-MT correction (see Fig. 4a) positive NotI pools were identified for both mutant variants in the initial screening. This screening process revealed three of the four pools, 1, 2 and 3 for both 5′NotI-MT and 3′NotI-MT screening showed some degree of NotI digestion, confirming the presence of corrected NotI sites within those pooled populations. The 1228 NotI digestion positive 3′NotI-MT and 5′NotI-MT pools were then screened further to identify the individual NotI corrected clones within each pooled population. Secondary screenings revealed three of the nine individual colonies of Pool 3 from the 3′NotI-MT reaction (Fig. 5b) and two of the nine individual colonies of Pool 1 from the 5′NotI-MT reaction (Fig. 5c) had been corrected.

**Figure 6:**
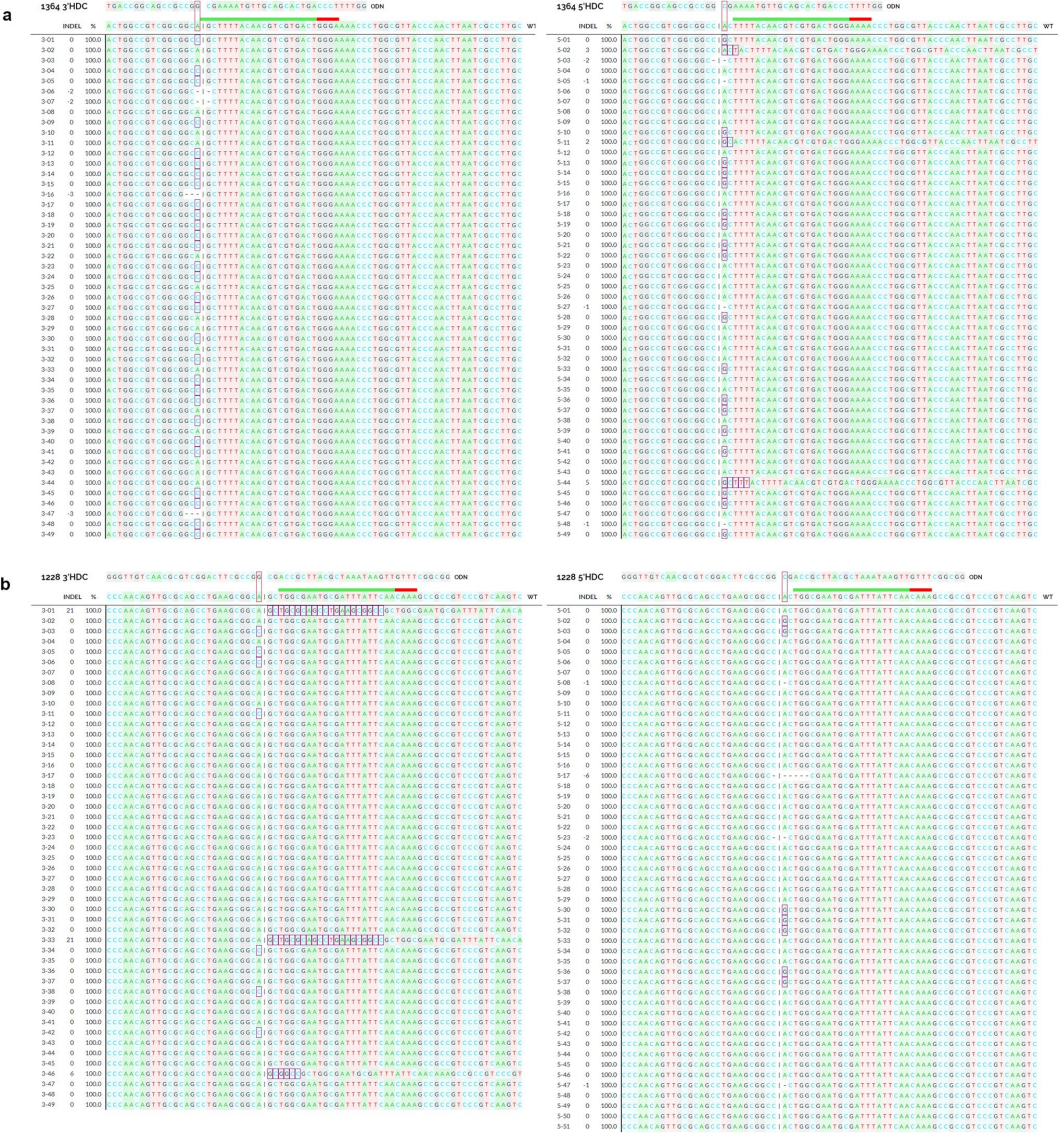
Sanger sequencing analysis of HDC repair outcomes. Individual clones were sequenced via Sanger sequencing to examine repair profiles of 3′NotI-MT (left) and 5′NotI-MT (right) at the (**a**) 1364 and (**b**) 1228 target sites. For each profile, a reference WT sequence of each NotI-MT plasmid is shown in alignment with the HDR correction template and the Cas12a target sites. The point mutations to be corrected are shown with the red boxes and the Cas12a cleavage sites are shown as a vertical dashed line. Aligned under the reference WT sequence, the individual repair profiles can be seen as insertions or modifications within purple boxes and deletions with dashed horizontal lines. Sanger sequencing analysis was done using DECODR (see “Materials and Methods”).

After confirming successful correction reactions were occurring through NotI digestion screening, we next wanted to investigate the repair profiles of the corrected 3′NotI-MT and 5′NotI-MT HDC reactions at the 1364 and 1228 sites. To visualize individual outcomes, we performed Sanger sequencing on randomly selected bacterial clones after transformation with plasmid recovered from HDC reactions. In total, 49 clones were sequenced for both 3′ and 5′ correction at the 1364 site, and 49 and 51 clones were sequenced for the 3′ and 5′ correction, respectively, at the 1228 site. Sequencing showed a varied of outcomes, including unedited WT, indels and precise correction, occurring simultaneously within the HDC reactions for 3′ and 5′-NotIMTs at both sites, 1228 and 1364. At the 1228 site, precise repair was seen in 7 of the 49 3′NotI-MT clones (14.3%) and 7 of the 51 5′NotI-MT clones (13.7%) (Fig. 6a). At the 1364 site, precise repair was seen in 29 of the 49 3′NotI-MT clones (59.2%) and 18 of the 49 5′NotI-MT clones (36.7%) (Fig. 6b).

**Figure 7:**
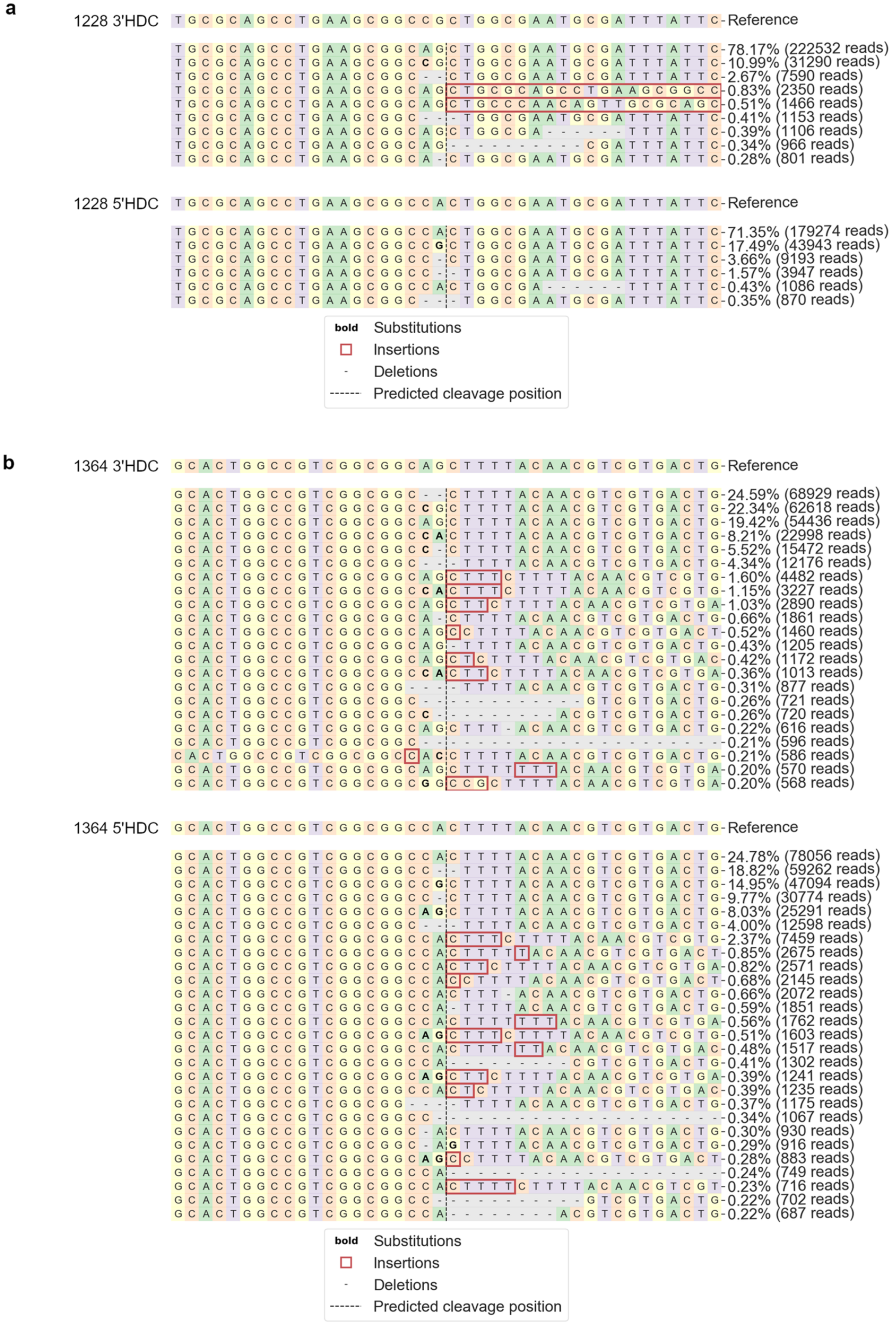
NGS analysis of HDC repair outcomes. DNA recovered after HDC reactions were sequenced via NGS to examine repair profiles of 3′NotI-MT (top) and 5′NotI-MT (bottom) at the (**a**) 1228 and (**b**) 1364 target sites. For each profile, a reference WT sequence of each NotI-MT plasmid is shown in alignment with the individual repair outcomes beneath. The predicted Cas12a cleavage site is shown as a vertical dashed line. The frequency at which each unique indel event was detected is shown to the right as a percentage of the total reads for each NGS reaction. Modifications are shown in bold, insertions within red boxes and deletions with dashed horizontal lines. NGS analysis was done using CRISPResso2 (see “Materials and Methods”).

After visualizing the variety of repair events after Sanger sequencing, we decided to performed Next Generation Sequencing (NGS) for each HDC reaction (Fig. 7a, b) to increase the depth of our on-target analysis. At the 1228 site (Fig. 7a), 11% and 17.5% precise correction was seen for the 3′ (top) and 5′ (bottom) reactions, respectively. At the 1364 site (Fig. 7b), 22.3% and 15% precise correction was seen for the 3′ (top) and 5′ (bottom) reactions, respectively. In addition to precise correction, indels at both cleavage sites were also quantified after HDC reactions. Overall, the frequency of indel events, meaning non-precise HDC and non-WT repair outcomes, at the 1228 site were consistent between the 3′ (10.8%) and 5′ (11.2%) reactions, but low compared to the frequency of indels seen at the 1364 site. In contrast, indel events made up the majority of the outcomes for both 3′ (58.2%) and 5′ (60.3%) HDC reactions at the 1364 site.

## Discussion

The evolution of CRISPR-Cas gene editing has given researchers a novel tool with which to disable malfunctioning genes or to correct single base mutations that are responsible for human disease. The simplicity of design, coupled with a high efficiency could enable a broad spectrum of therapeutic applications^29–31^. While significant advances in the success rate of gene editing continue to accumulate, by and large these positive outcomes involve directed gene knockout or the incorporation of exogenously added DNA fragments at specific target sites. Yet, efficient and precise correction of single base errors directed by CRISPR-Cas has remained elusive, and even when correction is achieved, it is often coupled with imprecision that would hinder clinical development.

In this work, we expand the foundational utility of a cell-free system that has already enabled us to elucidate details surrounding the mechanism of action of targeted deletion, gene replacement and fragment insertion^23–27^. This system has helped guide the design of optimal donor templates in the catalysis of HDR and helped elucidate reaction conditions that facilitate precise and efficient gene editing at a specified target. Now, we focused our attention on a system and strategy that centered on point mutation repair through the development of a model for Homolgy Directed Correction (HDC). To establish a robust system, we employed the Homology Directed Integration (HDI) reaction^25^ to generate selectable mutant plasmids containing mutated NotI restriction enzyme sites within the *lacZ* gene region of plasmid pHSG299. Each NotI-MT plasmid, termed 3′NotI-MT and 5′NotI-MT, at two locations along the *lacZ* gene, termed 1228 and 1364, contained a single base mismatch from the true NotI restriction enzyme site on either the 3′ and 5′ end of the Cas12a cleavage site.

At the 1364 site, a notable bias for precise single base correction is seen when the targeted mutation is positioned on the 3′ end of the staggered cut, as opposed to the 5′ end (see Fig. 2). Sanger sequencing analyses of individual clones (Fig. 6a, b) showed precise single base correction at the 1364 site occurring in 59.2% (29 of 49) and 36.7% (18 of 49) of the 3′NotI-MT and 5′NotI-MT clones, respectively. At the 1228 site, corrected 3′ and 5′ clones did not show a bias in overall correction efficiencies or distinct repair profiles, with 14.3% (7 of 49) and 13.7% (7 of 51) of the clones showing precise single base correction for 3′NotI-MT and 5′NotI-MT, respectively. The higher number of NotI digestion positive clones seen through the initial pooled colony screening for the 3′NotI-MT correction correlates with the higher number of precise corrections for the 3′ reaction at the 1364 site seen in the Sanger sequencing. When we expanded our analysis of on-site analysis with NGS sequencing, HDC reactions outcomes, including precise correction, indels and unchanged events, recapitulated the observations captured in the initial, smaller scale Sanger sequencing readout. The bias seen between the 3′ and 5′ correction at the 1364 site, but not at the 1228 site, as well as the notable increase in correction efficiency overall at the 1364 site compared to the 1228 site suggest that the influences driving precise correction are not necessarily correlated with either factor alone.

The functional relationships among DNA damage repair pathways and the corresponding repair process are undoubtedly complex and they can be influenced by many factors simultaneously. Broken chromosomal sites destined for repair act as substrates for distinct repair that could act competitively but be influenced by the availability of repair templates, their design and/or the environment in which the repair takes place. While targeted gene alteration via ssODN repair is thought to function primarily through HDR and nucleotide excision repair (NER), base excision repair (BER), microhomology mediated end joining (MMEJ) and SSA (single-stranded annealing) responses may also contribute^32–34^. The efficiency of correction for point mutations in the HDC system is lower than the efficiency we have previously seen for repair outcomes in other cell-free reactions, such as insertions^23^, gene segment replacements^24^ and HDI reactions^25^, an important correlation with in vivo studies of point mutation repair which also exhibit lower rates of correction efficiencies^1,32,35^. In other words, the HDC system described herein may be predictive of in vivo activity, strengthening further the correlation between cell-free in vitro and in vivo gene editing results. These studies establish a foundational model to design more precise and efficient tools and provide insight into factors that might overcome the low levels and imprecision of point mutation repair catalyzed by CRISPR-Cas.

## Materials and methods

### Cell-free extract preparation

HEK-293 cells (American Type Cell Culture, Manassas, VA) were cultured and 8 × 10^6^ cells were harvested and washed in cold hypotonic buffer (20 mM HEPES, 5 mM KCl, 1.5 mM MgCl_2_, 1 mM DTT and 250 mM sucrose). Cells were centrifuged and re-suspended in cold hypotonic buffer without sucrose, followed by incubation on ice for 15 min before being lysed by 25 strokes of a Dounce homogenizer. Cytoplasmic fraction of enriched cell lysate was incubated on ice for 1 h and then centrifuged for 15 min at 12,000 g, 4 °C. The supernatant was then aliquoted and stored at -80 °C. The concentration of cell-free extracts was determined using a Bradford assay.

### In vitro reaction conditions

In vitro HDI reactions, to generate NotI-MT plasmids, and HDC reactions, to correct the NotI-MT plasmids, both underwent cleavage reactions consisting of 500 ng (0.014 µM) of pHSG299 (Takara Bio Company, Shiga, Japan) or NotI-MT plasmid DNA and 10 pmol RNP in a reaction buffer (100 mM NaCl, 20 mM Tris–HCl, 10 mM MgCl2 and 100 µg/ml BSA) at a final volume of 20 µl. RNP complexes consisted of purified AsCas12a (AsCpf1) protein (Integrated DNA Technologies, Coralville, Iowa) and site-specific crRNA (Integrated DNA Technologies, Coralville, Iowa). Each reaction was incubated for 15 min at 37 °C, after which DNA was isolated from reaction mixtures and recovered using DNA Clean & Concentrator (Zymo Research, Irvine, CA). Secondary in vitro re-circularization reactions included DNA recovered from the initial cleavage reactions, 175 µg of cell-free extract supplemented with 400 cohesive end units of Quick T4 Ligase (New England Biolabs, Ipswich, MA), and 100 pmol of single-stranded donor DNA, *integration* template for HDI and *correction* template for HDC (Fig. 1), in a reaction buffer (20 mM TRIS, 15 mM MgCl_2_, 0.4 mM DTT and 1.0 mM ATP) at a final volume of 25 µl. Each reaction was then incubated for 15 min at 37 °C. Modified plasmid DNA from the final reaction mixture was then isolated and purified during spin column recovery.

### Transformation and selection

Modified plasmid DNA recovered from in vitro HDI and HDC reactions were transformed in 100 µL of DH5α Mix & Go! Cells competent *E. coli* (Zymo Research, Irvine, CA) incubated for 3 min on ice with the addition of 300 µl of SOC medium after which 100 µl of diluted cells were plated on media containing X-gal (Thermo Fisher Scientific, Wilmington, DE) and kanamycin and incubated overnight at 37 °C. Kanamycin resistant colonies were then selected for HDC screening.

### PCR amplification and NotI digestion screening.

Colony PCR amplification of the *lacZ* gene from selected bacterial colonies generated a 547 bp amplicon using PCR Primers (Integrated DNA Technologies, Coralville, Iowa) FWD 5′-GCTTCCGGCTCGTATGTTGTGTGG-3′ and REV 5′-GTTGGACGAGTCGGAATCGCAGA-3′. The PCR conditions involved an initial denaturation of template DNA at 94 °C for 2 min, cycle denaturation at 94 °C for 30 s, primer annealing at 60 °C for 1 min, and extension at 68 °C for 30 s for 35 cycles with a hold at 68 °C for 10 min. Each PCR contained either pooled or individually picked bacterial colonies, 10uM forward and reverse primers, PCR qualified water (Quality Biological Inc., Gaithersburg, MD) and *OneTaq* Quick-Load Master Mix (New England Biolabs, Ipswich, MA) in a total reaction volume of 50 µl. PCR products were purified using QlAquick PCR Purification Kit (Qiagen, Hilden, Germany). NotI digestion reactions were carried out using 250 ng of purified PCR amplicons, 10 × CutSmart buffer (New England Biolabs, Ipswich, MA), 20 units of NotI-HF (New England Biolabs, Ipswich, MA) and PCR qualified water (Quality Biological Inc., Gaithersburg, MD) at a final volume of 10 μl. Digestion reactions were incubated at 37 °C for 15 min, followed by the addition of 6 × gel loading dye (New England Biolabs, Ipswich, MA), loaded onto a 1% agarose gel and run for approximately 45 min at 100 V. Agarose gels were imaged with Azure c300 gel imager (Azure Biosystems, Dublin, CA).

### Sequencing analysis

HDC reaction outcomes were evaluated by either Sanger sequencing on a SeqStudio Genetic Analyzer (Applied Biosystems, Waltham, MA), with genetic outcomes visualized using DECODR^36^, or NGS by Amplicon-EZ paired-end sequencing (Azenta/Genewiz, South Plainfield, NJ), with genetic outcomes visualized using CRISPResso2^37^.

## Supplementary Information

Supplementary Information.

## Data Availability

The datasets generated during and/or analyzed during the current study are available from the corresponding authors on reasonable request.
