# Supplementary material for: Homology directed correction, a new pathway model for point mutation repair catalyzed by CRISPR-Cas
Source: Sci Rep. 2022 May 17;12:8132. doi: 10.1038/s41598-022-11808-2 (PMC9114366; doi:10.1038/s41598-022-11808-2)
Supplement: Supplementary file 1 — Supplementary Information. [file 41598_2022_11808_MOESM1_ESM.doc]

**Homology directed correction, a new pathway model for point mutation repair catalyzed by CRISPR-Cas**

Brett M. Sansbury^1^, Amanda M. Hewes^1^, Olivia M. Tharp^1,2^, Sophia B. Masciarelli^1,2^, Salma Kaouser^1^ and Eric B. Kmiec^1*^

^1^Gene Editing Institute, ChristianaCare Health System, Newark, Delaware

^2^ Department of Medical and Molecular Sciences, University of Delaware, Newark, Delaware

***Correspondence should be addressed to Eric B. Kmiec, Ph.D.**

Eric.B.Kmiec@christianacare.org

Gene Editing Insitute

550 S College Ave

Suite 100A, 2^nd^ Floor

Newark , Delaware 19713

# [www.christianacare.org/geneeditinginstitute](http://www.christianacare.org/geneeditinginstitute)

#
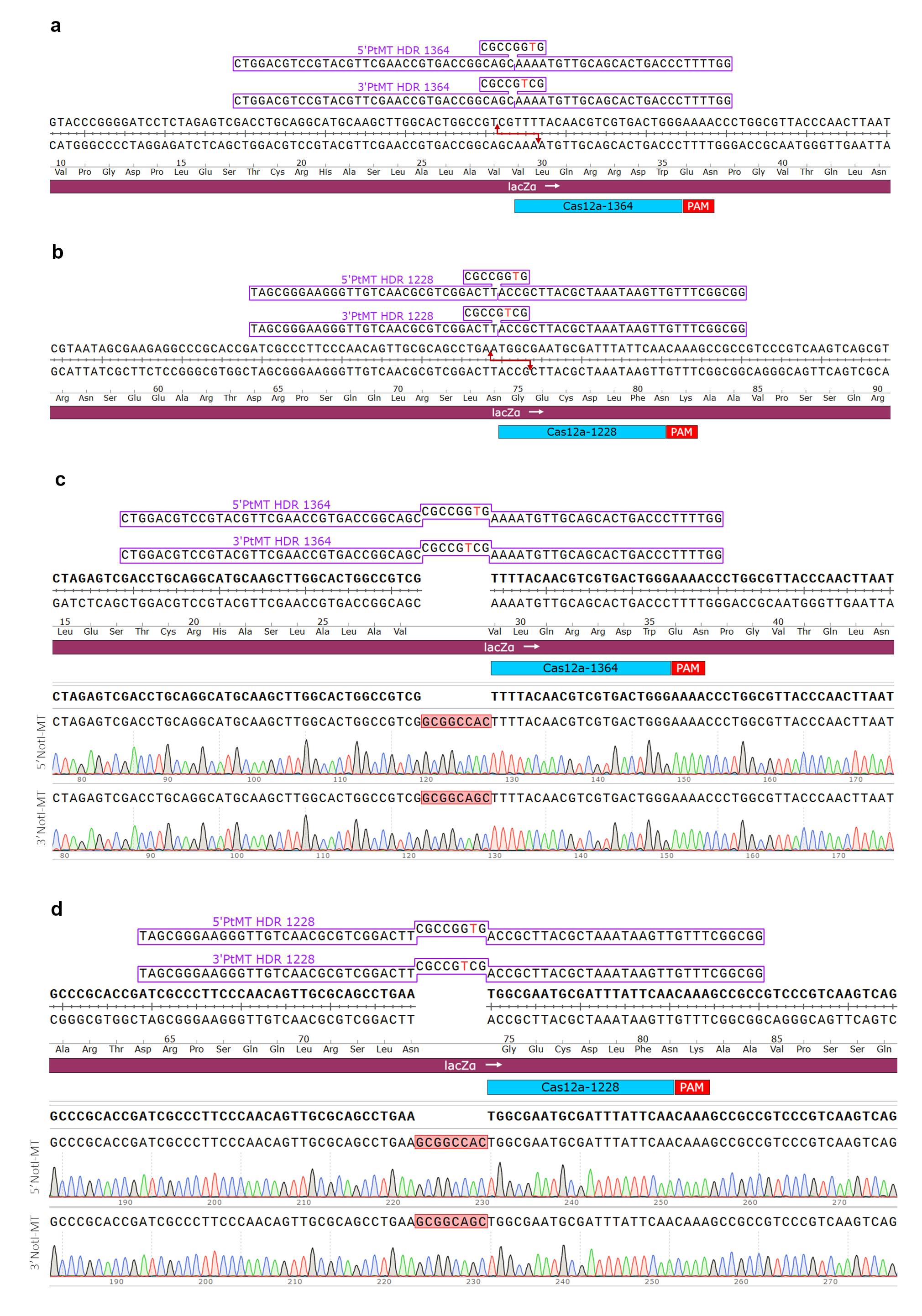


**Supplemental Figure 1. Experimental design of NotI-MT plasmids.** The Cas12a RNP cleavage sites for the (A) 1364 and (B) 1228 are shown along the *lacZ* gene as a staggered red arrow. The ssODNs used to generate 5’ (top) and 3’ (bottom) mutant plasmids through HDI reactions are shown above the indicated cut site. DNA sequencing was done to confirm the 5’ (top) and 3’ (bottom) NotI-MTs at the (C) 1364 and (D) 1228 sites after HDI reactions.


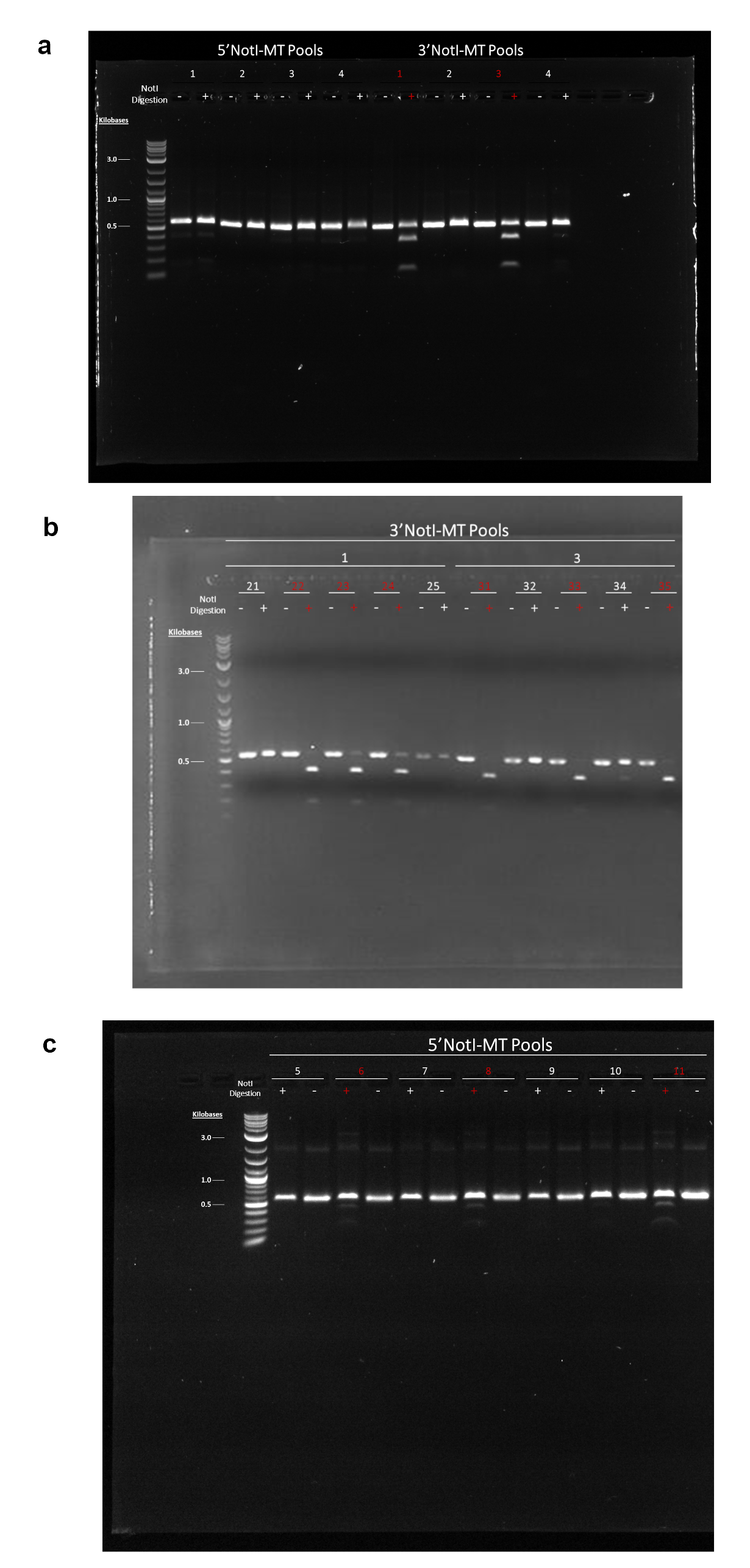


# Supplemental Figure 2. Original gels from Figure 4, pooled and single colony NotI digestions of 1364 NotI-MTs. (A) NotI digestions were done on four 5’ and 3’NotI-MT colony PCR pools consisting of 5 single colonies per pool. (B) NotI digestions were done on individual colonies from 3’NotI-MT colony PCR Pools 1 and 3. (C) NotI digestions were done on seven additional 5’NotI-MT colony PCR pools consisting of 5 single colonies per pool. Pools containing uncorrected NotI-MT DNA can be seen in lanes containing intact, undigested linear PCR amplicons as single bands. Pools containing corrected NotI-MT DNA can be seen in lanes with multiple bands after amplicon digestion are shown in red, with the upmost showing uncut, uncorrected PCR amplicon and the lower two showing successful NotI digested fragments


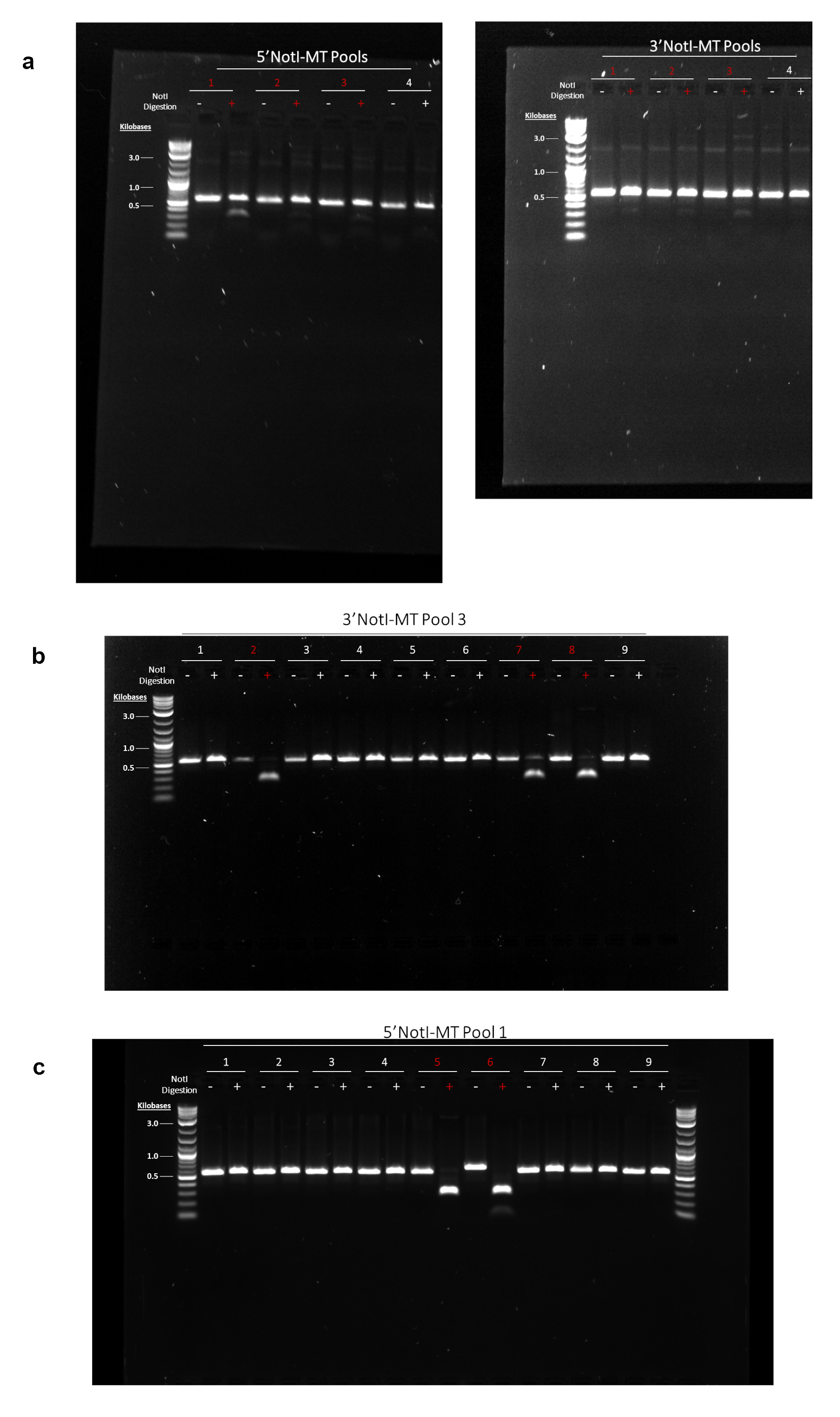


# Supplemental Figure 3. Original gels from Figure 5, pooled and single colony NotI digestions of 1228 NotI-MTs. (A) NotI digestions were done on four 5’ and 3’NotI-MT colony PCR pools consisting of 9 single colonies per pool. (B) NotI digestions were done on individual colonies from 3’NotI-MT colony PCR Pool 3 and (C) 5’NotI-MT colony PCR Pool 1. Pools containing uncorrected NotI-MT DNA can be seen in lanes containing intact, undigested linear PCR amplicons as single bands. Pools containing corrected NotI-MT DNA can be seen in lanes with multiple bands after amplicon digestion are shown in red, with the upmost showing uncut, uncorrected PCR amplicon and the lower two showing successful NotI digested fragments.
